# Supplementary material for: Changes in loneliness prevalence and its associated factors among Bangladeshi older adults during the COVID-19 pandemic
Source: PLoS One. 2022 Nov 4;17(11):e0277247. doi: 10.1371/journal.pone.0277247 (PMC9635750; doi:10.1371/journal.pone.0277247)
Supplement: Attachment — Submitted filename: Review response_R1.docx [file pone.0277247.s002.docx]

Response to Reviewers’ Comments - Loneliness is decreasing in Bangladeshi older adults during the COVID-19 pandemic, still the prevalence is high: A repeated cross-sectional study’s findings

Thank you for allowing us to revise and resubmit the manuscript and for the constructive comments. In the following table, we include the reviewers’ comments and outline our changes in responses to each comment. We have systematically incorporated reviewers’ comments and suggestions, which has enhanced the manuscript. We are confident that the revisions have improved the quality of our manuscript.

**Reviewer 1**

| **Comment/Suggestion** | **Authors’ Responses** |
| --- | --- |
| Thanks to the authors for addressing an important public health issue in the context of Bangladesh. Bangladesh is a poor developing country where this issue is often neglected but in developed countries it gets much importance. I am satisfied with the version, clear and concise. I personally know this team who are relentlessly working in different public health issues in Bangladesh that was unexplored earlier.  However, I have some comments regarding the paper which are mostly addressable. | Thanks very much for appreciating our effort. |
| What is the power of the study? | We determined the sample size considering a 90% power of test. Please see page 5 lines 141-142. |
| Why authors chose binary logistics regression since the scale gives you quantitative value, it would fit linear regression much better than only binary logistics regression? | Thanks very much for bringing this important issue to our notice. We agree to your point of conducting a linear regression analysis. However, our findings are particularly targeted towards the country's policymakers and public health practitioners with an aim to address the loneliness among this vulnerable population group. For this, we aimed to provide a cut-off value for the loneliness through which they can clearly understand the current situation of loneliness among the older adults and undertake interventions to address this. Therefore, we executed a logistic regression model using the cut-off as advised by Perissinotto et al. (2012).  Perissinotto CM, Cenzer IS, Covinsky KE. Loneliness in older persons: a predictor of functional decline and death. Archives of internal medicine. 2012;172(14):1078-84. |
| The conclusion of the abstract brought some generic comments do you have any specific suggestions how to reduce loneliness among older people in Bangladesh. | Many thanks for your thoughtful remarks. We have revised the conclusion of the abstract. Please see page 2-3 lines 64-68. |
| Most of the discussion written comparing to USA Canada UK they’re the highest developed countries, please establish the arguments with some Asian countries. | Thanks for raising this important concern. However, as we have mentioned in the Discussion section, there is a dearth of studies on COVID-19-related loneliness from low-income countries including those of Asian. Please see pages 15 lines 290-295. |
| Why authors doesn’t apply multilevel analysis since the data was collected from different heterogeneous divisions? It will suit better for reducing cluster effects. | Thanks very much for raising this important concern. We have used our pre-existing registry as a sampling frame for this study and from each administrative division, required number of households were selected using a simple random sampling technique from the list of eligible participants in the registry. For this, we opt to do simple logistic regression rather than any multilevel analysis such as GEE. |
| The title looks odd, please rework on the title. | Revised as suggested. |

**Reviewer 2**

| **Comment/Suggestion** | **Authors’ Responses** |
| --- | --- |
| This is a fairly standard analysis of two separate cross-sectional telephone surveys of loneliness among adults 60+ in Bangladesh that overlapped with the first and second wave or the COVID -19 in Bangladesh. Subjects were recruited from a pre-established household registry developed by the Aureolin Research, Consultancy and Expertise Development Foundation (ARCED). To be eligible to participate in the survey participants had to be a minimum of 60 years of age and free of common mental and cognitive disorders, not have a hearing disability or an inability to communicate. The study found the level of loneliness decrease between Time 1 and Time 2. The odds of loneliness were significantly higher people among who were without a partner, had a low income a who lived alone, had poor memory or concentration and suffered from non-communicable chronic disease. The odds of loneliness were significantly lower among individuals with formal education  While the manuscript and the findings interesting there are some concerns that the authors need to respond to: | We have addressed the concerns below: |
| 1) How representative are the samples of the Bangladeshi population? | While our study population covers all the eight administrative divisions of the country, the sampling frame for the study was constructed by merging the contact information of previously completed community-based studies conducted by ARCED Foundation. Moreover, amidst the pandemic, we had to conduct telephone interviews, and the sample may likely exclude those who don’t have telephone access. Therefore, our sample may not be representative of the entire older adults of Bangladesh. We have mentioned this in the limitation. Please see page 17, line 369-374. |
| 2) Are there censuses which could be used to compare the representativeness of the survey samples? | As we have mentioned, we had to conduct the study remotely amidst this pandemic. Therefore, the sampling frame for the study was constructed by merging the contact information of previously completed community-based studies conducted by the principal investigator’s institute. Therefore, we think the study sample cannot be compared with that of the nationally representative surveys such as the Bangladesh Demographic Survey or Household Income and Expenditure Survey. |
| 3) Where does the population registry that the survey samples are drawn come from? | A pre-existing registry, constructed based on previously completed community-based studies conducted by ARCED Foundation, served as the sampling frame for the current study and included households from all eight administrative divisions of Bangladesh. We have provided more detail of the registry and cited our previous articles where more details on this are presented. Please see page 5 lines 137-139. |
| 4) What is ARCED? Profit/non-profit? | It is a Bangladesh-based non-profit organisation. It is now elaborated. Please see page 5 lines 135-136. |
| 5) How specifically were the survey samples recruited from the existing registry? | In each administrative division, households were selected using a simple random sampling technique from the list of eligible participants in the registry, and subsequently, one eligible participant was interviewed from the selected household. Hence, the number of included households and respondents are equal. We have included this in the revised manuscript. Please see page 6 lines 148-151. |
| 6) Are participants paid? | The participant did not receive any compensation. Please see page 7 line 216. |
| 7) Since this is a telephone survey, what is the rate of telephone ownership in Bangladesh? Where cell/mobile phones or land lines used? | Thanks for raising your concern on this. We had to conduct telephone interviews, and, likely, the sample may not be representative of the entire older population of Bangladesh, explicitly excluding those who don’t have telephone access. We have added this information to the revised manuscript. Please see page 17, line 369-374.  Landline is not common in rural Bangladesh, therefore, none of the respondents had or were called over landlines, instead everyone was called overed cell/mobile phones. We have clarified this in the revised manuscript. Please see page 7 lines 186-187. |
| 8) Are the differences found in loneliness found between the initial and second cross-sectional surveys a result of difference in the composition of the samples at T1 and T2? | Thanks very much for this important question. We don’t think that the difference in loneliness is coming through the difference in the two samples as both of these samples were randomly selected from the same sampling frame. |
| Minor Points: |  |
| Page 6 1 st paragraph, line 2 - the UCLA Loneliness scale - as indicated by the rest of the paragraph this scale does not have Yes/No responses but rather has set of Likert types response. | Thanks very much for noting this. We have removed this. Please see page 6 line 159. |
| Page 6 2nd paragraph - line 1 - "literatureguided" - the words need to be separated.  " line 3 "rangpur' Needs to be capitalized | Thanks for noting this. Revised. Please see page 6 line 168 and line 170. |
| Page 15 last paragraph line 3 "anyrelated" the words need to be separated. | Revised. Please see page 16 line 329. |
